# External treatment of herbal medicine with tuina in congenital muscular torticollis: A protocol for systematic review and meta-analysis

**DOI:** 10.1097/MD.0000000000029035

**Published:** 2022-03-11

**Authors:** Eunjin Kim, Jungyoon Choi, Sang Yeon Min

**Affiliations:** a Department of Pediatrics of Korean Medicine, Korean Medicine Hospital, Dongguk University Bundang Medical Center, Republic of Korea; b Department of Pediatrics of Korean Medicine, Graduate School of Dongguk University, Republic of Korea; c Department of Pediatrics of Korean Medicine, Korean Medicine Hospital, Dongguk University Ilsan Medical Center, Republic of Korea.

**Keywords:** congenital muscular torticollis, external treatment, herbal medicine, meta-analysis, pediatrics, systematic reviews, tuina

## Abstract

**Background:**

This study is the protocol to evaluate the clinical evidence for external treatments using herbal medicine (ETHM) with tuina as a congenital muscular treatment (CMT) in pediatrics.

**Methods:**

Eleven databases will be searched until June 2022, without any language restrictions: four English databases (MEDLINE, Pubmed, EMBASE, and The Cochrane Central Register of Database of Controlled Trials), three Chinese databases (China National Knowledge Infrastructure, Chinese Scientific Journal Database, and Wan Fang Database), and four Korean electronic databases (Oriental Medicine Advanced Searching Integrated System, Korean Studies Information Service System, National Digital Science Links, and Research Information Sharing Service). This review will include randomized clinical trials (RCTs) of ETHM with tuina as an intervention versus the same tuina. All published RCTs for any ETHM for CMT will be included. Non-RCTs, RCT protocol, animal studies, case reports, reviews, and surveys will be excluded. The methodological quality assessment will be performed using the Cochrane risk of bias (ROBs). Review Manager version 5.4. will be used for the data synthesis and quantitative analysis.

**Results and discussions:**

The systematic review and meta-analysis will provide evidence for ETHM as a treatment of CMT. The evidence can help clinicians and patients recognize more effective therapeutic and safe inventions.

**INPLASY registration number:**

INPLASY202210017.

## 1 Introduction

Congenital muscular torticollis (CMT) is the third most frequent congenital musculoskeletal disorder in children after hip dysplasia and clubfoot.^[1]^ It is typically characterized by lateral head tilt and chin rotation to the contralateral side caused by unilateral contracture and shortening of the SCM (sternocleidomastoid muscle).^[2]^ The reported incidence of CMT is 1.3%^[3]^ to 16%^[4]^ in infants under 12 months. It frequently appears in males, firstborn babies, and the right lateral side.^[5,6]^

The clinical signs and physical examination based on the birth history are the easiest methods to diagnose CMT. The severity of CMT is classified into eight grades according to the age at which the asymmetry is first noticed, with or without mass, the range of muscle tightness degrees.^[7]^ Other pathologies, such as visual disturbances, neurological diseases, and vertebral anomalies (e.g., hemivertebrae, Goldenhar syndrome, and Klippel–Feil Syndrome) must be ruled out. The initial diagnosis and early treatment are important for preventing a long-term disfiguring complication, such as craniofacial asymmetric plagiocephaly, and compensatory scoliosis.^[8]^

There is no CMT therapeutic standardization. Therefore, collaborative inter-professional inventions are necessary. According to common procedures, passive range of motion (PROM) exercises are conducted for 12 weeks when a newborn has a neck mass, or an infant shows a head tilt.^[6]^ If the outcome is achieved, baby check-ups are needed until 12 months or when the child starts walking to assess reoccurrence and prevent atypical gross motor development.^[7]^ If the symptoms persist beyond 2 months without improvement, physical therapy to increase the infants’ active range of motion (AROM) and PROM is necessary for the first invention. Clinicians also use alternative and complementary therapies, such as manual therapy,^[9]^ soft neck braces, acupuncture, tuina,^[10]^ kinesiology taping,^[11]^ paraffin therapy, and external therapy of herbal medicine (ETHM) in combination. Surgery and botulinum neurotoxin therapy^[12]^ can be considered when the infant symptoms are not progressing as anticipated until 12 to 18 months.^[6]^

ETHM is the application of drugs to the surface of an illness, acupoint, or meridians.^[13]^ It has become widely used because of the high compliance for infants and no severe side effects.^[14]^ ETHM is absorbed^[15]^ transdermally in the form of creams, ointments, powders, and liquids,^[16]^ applied in the form of patch therapy^[17,18]^ or used as a medium during tuina treatment.^[19,20]^

Thus far, the ETHM performed with tuina for CMT effectively improves the infants’ symptoms. There are several studies on the efficacy and safety of tuina for CMT.^[10,21]^ On the other hand, few studies have examined the efficacy and safety of the ETHM plus tuina for CMT. This review systematically evaluated the effects of the ETHM with tuina compared to tuina alone in children aged 0 to 2 years. This could provide good evidence for clinicians.

## 2 Methods

This protocol followed the preferred reporting items for the systematic review and meta-analysis protocols (PRISMA-P) guidelines and the corresponding checklist.^[22]^ This study was registered on the internet platform of registered systematic review and meta-analysis protocol (Inplasy protocol 202210017) on January 5, 2022.

### 2.1 Eligibility criteria

#### 2.1.1 Types of studies

The reports on published RCTs of ETHM with tuina as an intervention will be compared with those on tuina alone. All types of ETHM with tuina for treating CMT in children under two years of age will be included. Non-RCTs, RCT protocol, animal studies, case reports, surveys, and reviews will be be excluded.

#### 2.1.2 Types of participants

Children aged 0 to 2 years will be included. They were diagnosed with any type of CMT. Torticollis patients caused by other diseases, such as skeletal torticollis, compensatory torticollis caused by atlantoaxial joint subluxation, visual impairment, hearing impairment, and neurotic torticollis caused by cervical muscle paralysis, will be excluded. Those with major organ function deterioration and complications, such as heart, liver, and kidney, will be also excluded.

#### 2.1.3 Types of interventions

The interventions of the experimental group will include tuina with any type of external application of traditional herbal medicine, with no limitation of the number of herbs, formulations (e.g., ointment, cream, powder, oil, patch, or liquid), dosages, or duration of the interventions. Studies on external application with heat stimulation or steam fumigation will be included. Tuina will include Chinese massage, manipulation, therapeutic massage, general massage, and relaxation.

#### 2.1.4 Types of comparator

The intervention of the controlled group will accept the same tuina therapy as the experimental group. Tuina that used a placebo (e.g., talcum powder) as a medium will be also included. Tuina combined with magnetic therapy or physiotherapy will be excluded.

#### 2.1.5 Types of outcome measure

1. Main outcomes
  (a) Total effective rate (TER): The total effective rate means the number of patients who improved their symptoms among the total participants.
  (b) The thickness of the mass in SCM
  (c) The cervical range of the motion of rotation or lateral flexion
2. Additional outcomes
  (a) Total symptom scores: Including muscle elasticity scores and SCM tightness degrees.
  (b) Relapse rates
  (c) Adverse effects

### 2.2 Information sources

#### 2.2.1 Data sources

The following databases will be searched from their founding date to June 2022, without any language restrictions: four English databases (MEDLINE, PubMed, Excerpta Medica database (EMBASE), and the Cochrane Central Register of Controlled Trials), three Chinese databases (China National Knowledge Infrastructure (CNKI), Chinese Scientific Journal Database (VIP), and Wan Fang Database), and four Korean medical databases (Oriental Medicine Advanced Searching Integrated System (OASIS), Korean Studies Information Service System (KISS), National Digital Science Links (NDSL), and Research Information Sharing Service [RISS]).

#### 2.2.2 Search strategy

Table 1 lists the search strategy used in Pubmed. A slight modification of the keywords will perform as a search strategy in other databases using the equivalent in each country's language.

**Table 1:** PubMed search strategy.

Number	Terms
#1	(“congenital muscular torticol∗”[Title/Abstract] OR “wry neck”[Title/Abstract] OR “twisted neck”[Title/Abstract] OR “cervical dystonia”[Title/Abstract])
#2	(“external treatment”[Title/Abstract] OR “external use”[Title/Abstract] OR “external application”[Title/Abstract] OR “topical use”[Title/Abstract] OR “topical treatment”[Title/Abstract] OR “dermal”[Title/Abstract] OR “skin”[Title/Abstract] OR “gel”[Title/Abstract] OR “ointment”[Title/Abstract] OR “cream”[Title/Abstract] OR “patch”[Title/Abstract] OR “oil”[Title/Abstract] OR “powder”[Title/Abstract] OR “liquid”[Title/Abstract])
#3	(“herb”[Title/Abstract] OR “herbal medicine”[Title/Abstract] OR “plant”[Title/Abstract] OR “plant extract”[Title/Abstract] OR “decoction”[Title/Abstract] OR “Traditional medicine”[Title/Abstract] OR “traditional Chinese medicine”[Title/Abstract] OR “traditional Korean medicine”[Title/Abstract] OR “Kampo medicine”[Title/Abstract])
#4	(“newborn∗”[Title/Abstract] OR “child∗”[Title/Abstract] OR “baby”[Title/Abstract] OR infant∗“[Title/Abstract] OR ”youth“[Title/Abstract] OR ”pediatric∗“[Title/Abstract] OR ”paediatric∗“[Title/Abstract] OR ”toddler∗"[Title/Abstract])
#5	#1 AND #2 AND #3 AND #4

### 2.3 Study selection and data extraction

#### 2.3.1 Study selection

**Figure 1:**
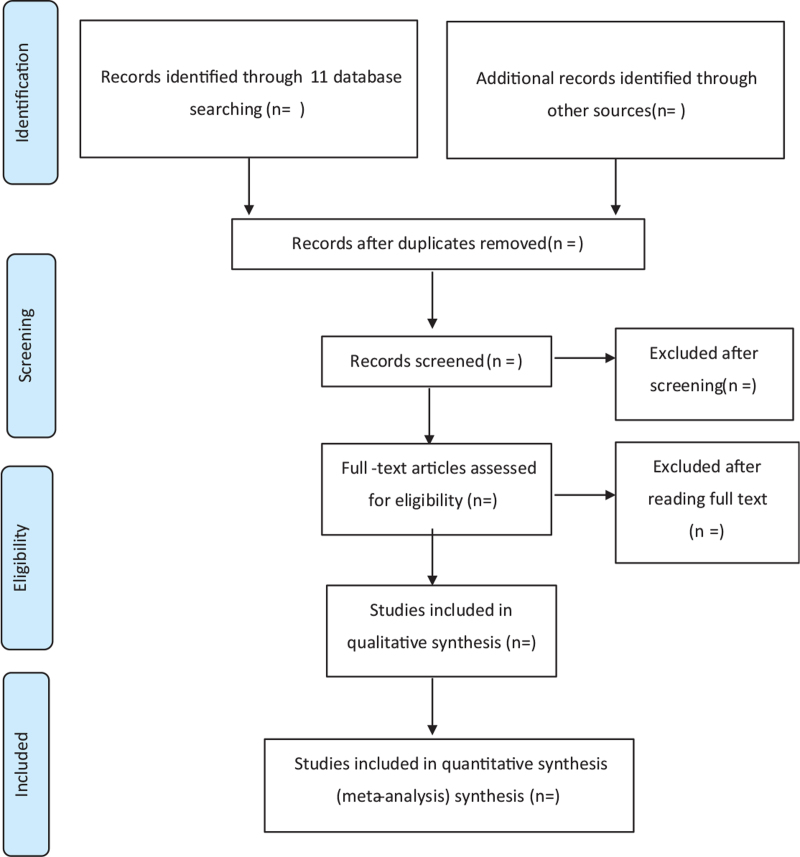
PRISMA flow chart of the search process. PRISMA = Preferred Reporting Items for Systematic Reviews and Meta-Analyses.

Two independent authors (EJK and JYC) will read the literature by screening the title and abstracts according to pre-defined criteria and conducted a crosscheck. Figure 1 presents a flowchart of the search process. In the case of disagreement, the full text will be reviewed and resolved through discussion among all the authors (EJK, JYC, and SYM).

#### 2.3.2 Data extraction

All articles will be read by two independent reviewers (EJK and JYC). The data will be extracted according to pre-designed excel forms: basic study design information (year of publication, the name of the first author, type of RCT, and sample size), patient information (sex, age, type of CMT, and birth history), interventions and controls (type of ETHM with tuina), treatment period, outcome measurement (total effective rate, the thickness of the mass in SCM, the cervical range of the motion of rotation or lateral flexion, total symptom scores, relapse rates, and adverse effects), and follow-up period.

### 2.4 Assessment of the risk of bias

Quality assessments will be performed using the risk of bias (Rob) from the Cochrane Handbook^[23]^ for Systematic Reviews of Interventions, including sequence generation, allocation concealment, blinding, incomplete outcome data, selective outcome reporting, and other sources of bias. Two dependent authors (EJK and JYC) will summarize the assessments and categorize the included studies into three levels of bias (low, unclear, and high risk of bias).

### 2.5 Data synthesis

Two authors (EJK and JYC) independently will synthesize the data by Review Manager 5.4 software. For the dichotomous outcomes, the data will be summarized using the risk ratio (RR) with 95% confidence intervals (CIs). And for the continuous outcomes, standard mean difference (SMD) or mean difference (MD) with 95% CIs will be used to measure outcomes.

### 2.6 Assessment of heterogeneity

For heterogeneity test among literature trials, it will be evaluated by the *I*^2^ and Chi-squared test statistics. If *I*^2^ > 50%, we consider the included studies have high heterogeneity and use a random-effect model for pooling data across studies. And if *I*^2^ < 50%, we believe that there is low heterogeneity and fixed-effect model will be used for data analysis.

### 2.7 Assessment of reporting biases

If there are more than 10 studies in the meta-analysis, the funnel plot will be assessed to evaluate reporting biases.

### 2.8 Subgroup and sensitivity analysis

Subgroups analysis will be performed when meta-analysis showed significant heterogeneity and there are sufficient data: such as age, birth history, and type of ETHM. The robustness of the results will be tested through sensitivity analysis by excluding low-quality trials, small sample trials, and high bias risks. If we cannot explain the significant heterogeneity, we will perform the descriptive systematic reviews.

### 2.9 Grading the quality of evidence

Two authors (EJK and JYC) independently will evaluate the quality of the evidence of all literature outcomes by using 4 level (”high,” “moderate,” “low,” and “very low”) Grading of Recommendations Assessment, Development, and Evaluation (GRADE) rating standards.

### 2.10 Ethics

Ethical approval was not necessary, because this study only evaluated the published literature.

## 3 Discussion

CMT is characterized by an asymmetry of the head and neck posture caused by the thickness and shortening of the sternocleidomastoid muscle (SCM). Three types of CMT have been categorized. It is presented most in order of an SCM mass (SMT), muscular torticollis (MT), and postural torticollis (POST). SMT, the most severe type of CMT, manifests as a palpable intramuscular fibrotic thickening in the SCM with restricted PROM. The condition may be detected at birth or during the first 4 weeks by a doctor, midwife, or parents.^[24]^ MT also has a limitation in cervical PROM due to tightness in the SCM without a mass or tumor.^[2]^ Infants with POST, the mildest type, have a positional preference due to a weakness of unilateral SCM. It presents without a muscle mass and PROM limitation.^[25]^ The etiology of CMT remains uncertain. It can be caused by a higher compression of the fetus toward the end of pregnancy, intrauterine malposition, traumatic deliveries, such as forceps and suction cups,^[26]^ and the length of the infants at birth are 51.3 cm or more.^[7]^ CMT can affect developmental motor delay, neurological dysfunction, or musculoskeletal problems.^[27]^

Complementary therapies commonly treat CMT include tuina, acupuncture, and moxa therapy in East Asia. Tuina is the most widely used invention in combination with paraffin therapy,^[28]^ herbal fumigation therapy,^[29,30]^ and external therapy of herbal medicine (ETHM). The pharmacological action of ETHM treats diseases by flowing the meridians smoothly, balancing yin and yang, and circulating the blood.^[31]^ In addition, herbal medicine promotes wound healing, suppresses scar formation,^[32]^ and treats chronic diseases^[33]^ by regulating cytokines. ETHMs are commonly used for musculoskeletal disorders, such as osteoarthritis^[34]^ and external humeral epicondylitis^[35]^ as well as acne vulgaris^[36]^ and asthma,^[14]^ but the key problems of safety and low absorption efficiency remain.^[37]^ There are insufficient clinical and literature studies and no systematic study of using ETHMs for CMT. Accordingly, the purpose of this study is to assess the efficacy and safety of ETHMs plus tuina in CMT and provide objective evidence for future research.

This study has some limitations. First, it is difficult to limit the formulation, method, frequency, and dosage of ETHMs, so there may be heterogeneity. Second, some location bias may occur using ETHMs and tuina as an intervention, which is mainly used in East Asia. Third, documents not been published in the electronic database will not be included, and the evaluation will be only among published studies. On the other hand, this study can provide great evidence for treating CMT infants clinicians considering integrated treatments.

## Author contributions

**Conceptualization:** Eunjin Kim, Jungyoon Choi.

**Data curation:** Eunjin Kim, Jungyoon Choi.

**Formal analysis:** Eunjin Kim, Jungyoon Choi.

**Investigation:** Eunjin Kim, Jungyoon Choi, Sang Yeon Min.

**Methodology:** Eunjin Kim, Jungyoon Choi.

**Project administration:** Sang Yeon Min.

**Resources:** Eunjin Kim, Jungyoon Choi.

**Software:** Eunjin Kim, Jungyoon Choi.

**Supervision:** Sang Yeon Min.

**Validation:** Sang Yeon Min.

**Visualization:** Eunjin Kim, Jungyoon Choi.

**Writing – original draft:** Eunjin Kim, Jungyoon Choi.

**Writing – review & editing:** Eunjin Kim, Jungyoon Choi, Sang Yeon Min.
